# Protective effects of Danshen injection against erectile dysfunction via suppression of endoplasmic reticulum stress activation in a streptozotocin-induced diabetic rat model

**DOI:** 10.1186/s12906-018-2414-3

**Published:** 2018-12-27

**Authors:** Yong Zhang, Jian Chen, Hua Ji, Zhang-Gang Xiao, Peiqiang Shen, Lin-Hao Xu

**Affiliations:** 1 Department of Urology, The Second Affiliated Hospital, School of Medicine, Zhejiang University, Hangzhou, 310009 Zhejiang China; 2 Faculty of Basic Medicine, Hangzhou Medical College, |No. 481 Binwen Road, Binjiang District, Hangzhou, 310053 Zhejiang China; 3 Laboratory of Molecular Pharmacology, Department of Pharmacology, School of Pharmacy, Southwest Medical University, Luzhou, 646000 Sihuan China; 4 Key Laboratory of Medical Electrophysiology, Ministry of Education, School of Pharmacy, Southwest Medical University, Luzhou, 646000 Sihuan China; 5 Research and Development Center of Chiatai Qingchunbao, Hangzhou, Zhejiang 310000 People’s Republic of China

**Keywords:** Diabetes, Erectile dysfunction, Danshen injection, Endoplasmic reticulum stress, Oxidative stress

## Abstract

**Background:**

Erectile dysfunction (ED) is a common complication of diabetes. This study aimed to explore the beneficial effect of Danshen injection on ED in a streptozotocin (STZ)-induced diabetic rat model and the underlying mechanism.

**Methods:**

The diabetic rat model was established by an intraperitoneal injection of 60 mg/kg STZ in male Sprague-Dawley rats. The diabetic rats were intraperitoneally injected with Danshen solution (0.5 or 1 mL/kg/day) or the same volume of saline for 6 weeks. Age-matched rats served as controls. After 6 weeks, erectile function and histological morphology of the corpora cavernosum were assessed. Oxidative stress indicators, including superoxide dismutase (SOD) activity, malondialdehyde (MDA) content, and reactive oxygen species (ROS) levels, were measured in penile tissues. The expression levels of glucose-regulated protein 78 (Grp78), growth arrest and DNA damage-inducible gene 153 (GADD153/CHOP) were determined by immunohistochemistry, immunoblotting, and RT-PCR. Apoptosis was detected by a TUNEL assay.

**Results:**

The erection times of diabetic rats were significantly less than those of control rats. Danshen injection could improve erectile function via increased erection times. Danshen injection was also found to ameliorate the morphological abnormalities of the corpora cavernosum, to reduce the number of apoptotic cells, and to suppress caspase-3 activation in penile tissue, accompanied by downregulation of the endoplasmic reticulum stress biomarkers Grp78 and CHOP. Danshen injection could increase SOD activity as well as reduce ROS and MDA levels in diabetic rats, indicating suppression of oxidative stress.

**Conclusion:**

Danshen injection could rescue diabetes-associated ED, possibly via suppressing the oxidative stress and endoplasmic reticulum (ER) stress-induced apoptosis pathways.

## Background

Erectile dysfunction (ED) is defined as the inability of a man to obtain and maintain a persistent erection during sexual intercourse. ED has a strong unfavorable impact on the quality of life of patients. Diabetes mellitus (DM) is a chronic disease with multiple complications. It is estimated that there are 415 million adult diabetics worldwide and an additional 318 million adults with impaired glucose tolerance [1]. There is a close relationship between DM and ED. It has been reported that diabetic men are three times more likely to develop ED than nondiabetic men [2] and that the prevalence of ED in patients with DM is approximately 75.2% in China [3].

Hyperglycemia may cause multifactorial metabolic disorders in DM patients. Thus, the pathogenesis of ED in DM patients may involve various pathophysiological factors, including oxidative stress, production of advanced glycation end-products, activation of the polyol pathway, deficiency of nerve growth factor, dysfunction of protein kinase C, and abnormalities in the transforming growth factor-β1 signaling pathway [4–6]. Recently, an increase in the number of apoptotic cells was observed in erectile tissue of both DM patients and animal models [7–9], whereas inhibition of apoptosis activation could restore erectile function, suggesting that apoptosis might play an important role in the pathogenesis of ED. However, the exact mechanisms are still elusive.

The endoplasmic reticulum (ER) is an organelle that is responsible for protein synthesis and folding as well as the delivery of apoptotic signals [10]. It is susceptible to various types of injury. It is well known that redox homeostasis has a critical role in protein synthesis and folding. The oxidizing environment in the ER lumen facilitates the formation of non-native disulfide bonds, which can lead to protein misfolding or protein inactivation [11]. Therefore, hyperglycemia-induced oxidative stress or an oxidizing environment may trigger ER stress, which was demonstrated in our previous study [12].

It has been reported that ER stress-induced apoptosis can result in various forms of cellular dysfunction and even cell death in the hippocampus and myocardial tissues [13, 14]. However, few studies have been conducted to investigate the presence of ER stress-induced apoptosis in the corpus cavernosum during the progression of diabetes. We speculate that ER stress-induced apoptosis is involved in the pathogenesis of diabetic ED, while amelioration of ER stress and the resultant apoptosis could be a potential therapeutic strategy for the treatment of ED in diabetic patients.

Danshen, a traditional Chinese herb, is the dried root of the plant *Salvia miltiorrhiza Bunge*. In a clinical trial, Danshen injection has been demonstrated to cure ED and increase the low-level arterial blood supply in corpus cavernosum tissue of ED patients [15]; however, the underlying mechanism has not been fully investigated. In addition, a recent study has revealed that salvianolic acid B, the main bioactive component in the root of *S. miltiorrhiza*, has a vascular protective effect in diabetes via attenuating cell apoptosis, which might be associated with amelioration of oxidative stress [16]. Indeed, the effect of Danshen injection on suppressing the oxidative stress level has been confirmed in previous studies [17, 18]. Therefore, we hypothesized that Danshen injection could improve erectile function via reduction of the oxidative stress level and inhibition of cell apoptosis.

In this study, we investigated whether Danshen injection exerts a protective role in maintaining erectile function in a diabetic rat model through suppression of ER stress-induced apoptosis in the corpus cavernosum.

## Methods

### Animals

A total of 40 six-week-old male Sprague-Dawley rats (160–180 g) were purchased from the Experimental Animal Center of Zhejiang University, and they were divided into four groups using a randomized complete block design (*n* = 10 each), namely the control group, diabetes group, and two Danshen injection groups (0.5 or 1 mL/kg). The rats were housed under specific pathogen-free conditions with controlled lighting (12 h per day) and temperature (21 ± 2 °C), fed standard laboratory food, and allowed water ad libitum at the animal laboratories of Hangzhou Medical College. Two rats were placed in one cage. All procedures in this study were carried out in accordance with the National Institutes of Health Guide for the Care and Use of Laboratory Animals. The experimental protocol was approved by the Ethics Committee of Hangzhou Medical College.

### Danshen injection

Danshen, with aqueous extracts of *Radix S. miltiorrhiza* as its active components, was purchased from Chiatai Qingchunbao Pharmaceutical Co., Ltd. (Hangzhou, Zhejiang, China) and were identified and authenticated by Peiqiang Shen, associate principal scientist, research and development center of Chiatai Qingchunbao pharmaceutical Co., Ltd. The voucher specimen of this material (NO.2016-9-18) has been deposited in Chiatai Qingchunbao Pharmaceutical Co., Ltd.. Its chemical and metabolic fingerprints were determined by high-performance liquid chromatography, as described previously [17].

### Rat model of diabetes

Thirty rats were fasted for 12 h before establishment of the diabetic rat model by a single intraperitoneal injection of streptozotocin (STZ, 60 mg/kg, Sigma-Aldrich Corporation, St. Louis, MO, USA). Two days after the STZ injection, the plasma glucose level was measured with a blood glucose analyzer and strips (Abbott Laboratories, Chicago, IL, USA), and the rats with a glucose level > 16 mM were regarded as diabetic. The remaining ten rats served as controls and received an injection of 0.9% saline after fasting for 12 h. Then, the diabetic rats were intraperitoneally administered with an equal volume of Danshen solution (0.5 or 1 mL/kg, based on a human equivalent dose, Danshen injection groups) or 0.9% saline (diabetes group) once a day for 6 weeks. The injection volume was 1 mL for each rat (diluted by 0.9% saline). The rats in the control and diabetes groups intraperitoneally received 1 mL of 0.9% saline.

### Assessment of erectile function

After 6 weeks of Danshen or saline injections, apomorphine (80 μg/kg, Sigma-Aldrich, Darmstadt, Germany) dissolved in saline containing 0.5 mg/kg vitamin C was injected subcutaneously to assess the erectile function of rats. On examination, all rats were maintained in a quiet environment with dim lighting, and the times and duration of penile erections were recorded within 30 min. Erection was defined as observation of penile hyperemia and the glans.

### Histochemistry and immunohistochemistry

After assessment of erectile function, five rats from each group were anaesthetized by an intraperitoneal injection of 1% pentobarbital sodium salt (30 mg/kg, Sigma-Aldrich, Darmstadt, Germany). Then, these rats were perfused with 50 mL of saline, followed by 200 mL of 4% paraformaldehyde solution for histological preservation, immunohistochemistry and TUNEL staining. After fixation for 24 h, the penile tissue was embedded in paraffin and cut into two sets of 4 μm-thick sections.

One set was used for routine histopathological observations with hematoxylin-eosin staining, while the other set was used to determine the expression of target proteins by immunohistochemistry.

For immunohistochemistry, the sections were deparaffinized, rehydrated with gradient alcohol solutions, immersed in 10 mM citric acid (pH 6.0), and washed with distilled water, in sequence. The sections were then treated with 3% hydrogen peroxide to block endogenous peroxidase activity, followed by incubation with primary antibodies (rabbit anti-rat Grp78, 1:300; rabbit monoclonal mouse anti-rat CHOP, 1:100; both from Cell Signaling Technology, Danvers, MA, USA) overnight at 4 °C. Then, the sections were incubated with polymerase adjuvant for 20 min and subsequent avidin-biotin-peroxidase complex solution (BOSTER Biological Technology, Wuhan, China) for 30 min at room temperature. Finally, the sections were visualized by diaminobenzidine staining and hematoxylin counterstaining, and sealed. Each section was scanned by a Pannoramic MIDI (3DHISTECH, Hungary). The staining score for cells in penile tissue was determined under high (400×) magnification by a panoramic view, including cells in the cavernous tissue, as described previously [19]. The intensity of immunoreactivity was evaluated as 0, negative; 1+, weak; 2+, moderate; and 3+, strong. The H-score was calculated according to the following formula: H-score = (percentage of cells of weak intensity × 1) + (percentage of cells of moderate intensity × 2) + (percentage of cells of high intensity × 3). The intensity of staining was quantified by the automatic recognition software Quant Center. A digital microscope (Zeiss microscope axiophot 2, Zeiss, Germany) was used to observe positive cells at 400× magnification.

### Real-time quantitative reverse transcription polymerase chain reaction (qRT-PCR) analysis

The remaining rats (another five in each group) were also euthanized by an intraperitoneal injection of 2% pentobarbital sodium salt (60 mg/kg, Sigma-Aldrich, Darmstadt, Germany). The penile tissues were collected and stored in liquid nitrogen for detecting qRT-PCR, reactive oxygen species (ROS), superoxide dismutase (SOD) activity and malondialdehyde (MDA) content.

Total RNA was extracted from samples using the Trizol reagent kit (Invitrogen, Carlsbad, CA, USA), according to the manufacturer’s instructions. The RNA concentration was measured on a spectrophotometer, and 1 μg of total RNA was reversely transcribed to complementary DNA (cDNA) using a Transcriptor First Strand cDNA Synthesis Kit (TaKaRa, Japan). For qRT-PCR analysis, the reaction mixture (20 μL) consisted of 2 μL of cDNA, 10 μL of SYBR® Premix Ex Taq™ (TaKaRa, Japan), 1 μL of 2.5 U/μL Taq DNA polymerase, 1 μL of 10 pmol/μL Grp78 or CHOP primer (Invitrogen, USA), and 6 μL of ddH_2_O. The thermal cycling conditions were an initial denaturation at 94 °C for 3 min; amplification by 40 rounds of 94 °C for 10 s, 60 °C for 30 s, and 72 °C for 30 s; and a final extension at 72 °C. The primers used included the housekeeping gene glyceraldehyde-3-phosphate dehydrogenase (GAPDH), forward: 5′-GGTGGACCTCATGGCCTACAT-3′, reverse: 5′-GCCTCTCTCTTGCTCTCAGTATCCT-3′ as an internal control; Grp78, forward: 5′-TGATGCCCAGCGACAAGC-3′, reverse: 5′-CGCCACCCAGGTCAAACA-3′; and CHOP, forward: 5′-CTGCTCCTTCTCCTTCATGC-3′, reverse: 5′-AGCAGAGGTCACAAGCACCT-3′.

### Western blot analysis

Total proteins were extracted from penile tissues using ice-cold radioimmunoprecipitation assay buffer (Thermo Fisher Scientific, Waltham, MA, USA). The total protein concentration was measured by using a bicinchonic acid Protein Assay Reagent Kit (Pierce Biotechnology, Rockford, IL, USA). Total protein (30 μg) of each sample was transferred to a polyvinylidene difluoride membrane (Millipore Corporation, Billerica, MA, USA) at 18 V for 1 h by the semi-dry transfer method. The membranes were blocked with 5% nonfat dry milk at 37 °C for 1 h and incubated with the relevant primary antibody (monoclonal anti-CHOP, polyclonal activated caspase-3, or polyclonal Grp78 from Cell Signaling Technology; β-actin from Bio-Rad Laboratories) at 4 °C overnight. After washing three times with Tris-buffered saline-Tween 20, the blots were incubated with fluorescent-labeled secondary antibody (LI-COR Biotechnology, Lincoln, NE, USA) for 1 h at room temperature. Reactions were visualized using an Odyssey scanner. The density of the corresponding bands was analyzed quantitatively by the Odyssey infrared imaging system (LI-COR GmbH, Germany), with a resolution of 169 μm and corrected by reference to the value for β-actin.

### Reactive oxygen species (ROS) assay

Penile tissue was weighed and homogenized to obtain homogenate, which was then centrifuged at 6000 rpm and 4 °C for 15 min. A total of 5 μL of supernatant was mixed with 55 μL of HEPES (0.02 M) and 90 μL of fresh 2′,7′-dichlorofluorescein-diacetate (20 μM, Sigma-Aldrich, St. Louis, MO, USA) in a black, flat-bottom 96-well plate and incubated at 37 °C for 30 min. The fluorescence was quantified by a plate reader (Synergy Mx, BioTek, Winooski, VT, USA) at an excitation wavelength of 485 nm.

### TUNEL staining

After preparation of 4-μm-thick sections, the apoptotic cells in the penile tissues were evaluated by TUNEL staining (Abcam, Cambridge, MA, USA) on five consecutive sections using an ApopTag1 kit (Millipore Corporation), according to the manufacturer’s instructions.

### Superoxide dismutase (SOD) activity and malondialdehyde (MDA) content

The frozen penile tissue was rinsed with 0.9% saline. Homogenates were centrifuged at 10,000×*g* and 4 °C for 20 min, and the protein content in the supernatant was measured by the Lowry method, with bovine serum albumin as the standard. SOD activity and MDA content in the penile tissue were detected by spectrophotometry using commercially available kits (Jiancheng Bioengineering Institute, Nanjing, China).

### Statistical evaluation

All data were expressed as the mean ± standard error of the mean (S.E.M.). The inter-group differences were determined by one-way analysis of variance followed by the Bonferroni post hoc test for multiple comparisons. A *P*-value < 0.05 was considered significant.

## Results

### Danshen injection restored the body weight without affecting the blood glucose level

**Fig. 1:**
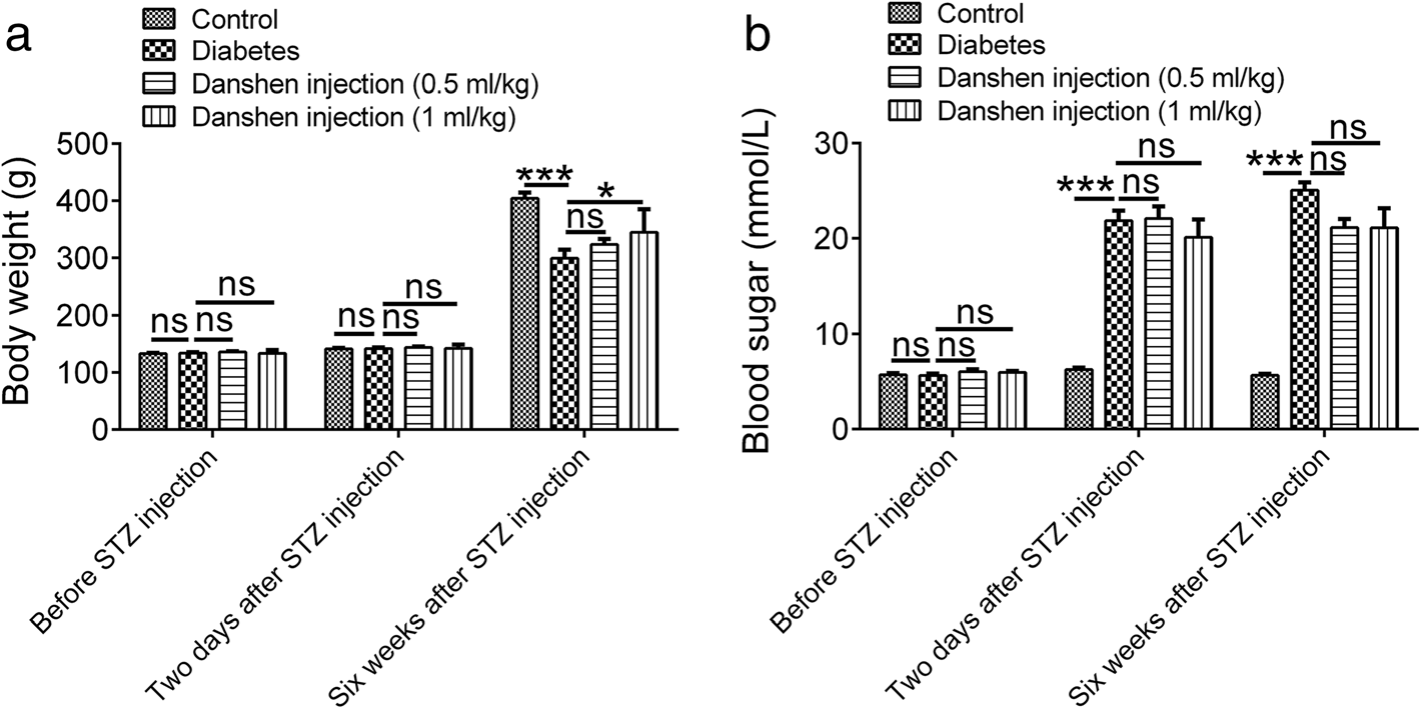
Danshen injection restored the body weight of diabetic rats without affecting the blood glucose level. **a** Body weight; **b** blood glucose levels before and at 2 days and 6 weeks after treatment. Data are presented as the mean ± standard error of the mean (S.E.M.) for ten rats per group. *P < 0.05; ***P < 0.001; ns, not significant

There was no significant difference in the body weight before STZ injection among groups and 2 days after STZ injection (*P* > 0.05, Fig. 1a). However, at 6 weeks after treatment, the body weight in the diabetes group was 299.26 ± 14.91 g, which was only about 74% of that of the control group (404.44 ± 14.91 g). Danshen injection (0.5 or 1 mL/kg) could restore the body weight of the diabetic rats (329.97 ± 8.56 g; 344.60 ± 12.90 g). In particular, the body weight in the Danshen injection (1 mL/kg) group was significantly higher than that of the diabetes group at 6 weeks after STZ injection (*P* < 0.05, Fig. 1a).

The blood glucose levels were similar among groups before STZ injection. However, they were significantly elevated at 2 days after STZ injection and persistently increased during the next 6 weeks (*P* < 0.001, Fig. 1b). Diabetic rats treated with Danshen injection (0.5 or 1 mL/kg) exhibited a similar trend in blood glucose levels compared to those in the diabetes group (*P* > 0.05, Fig. 1b).

### Danshen injection could improve erectile function via increasing erection times

**Fig. 2:**
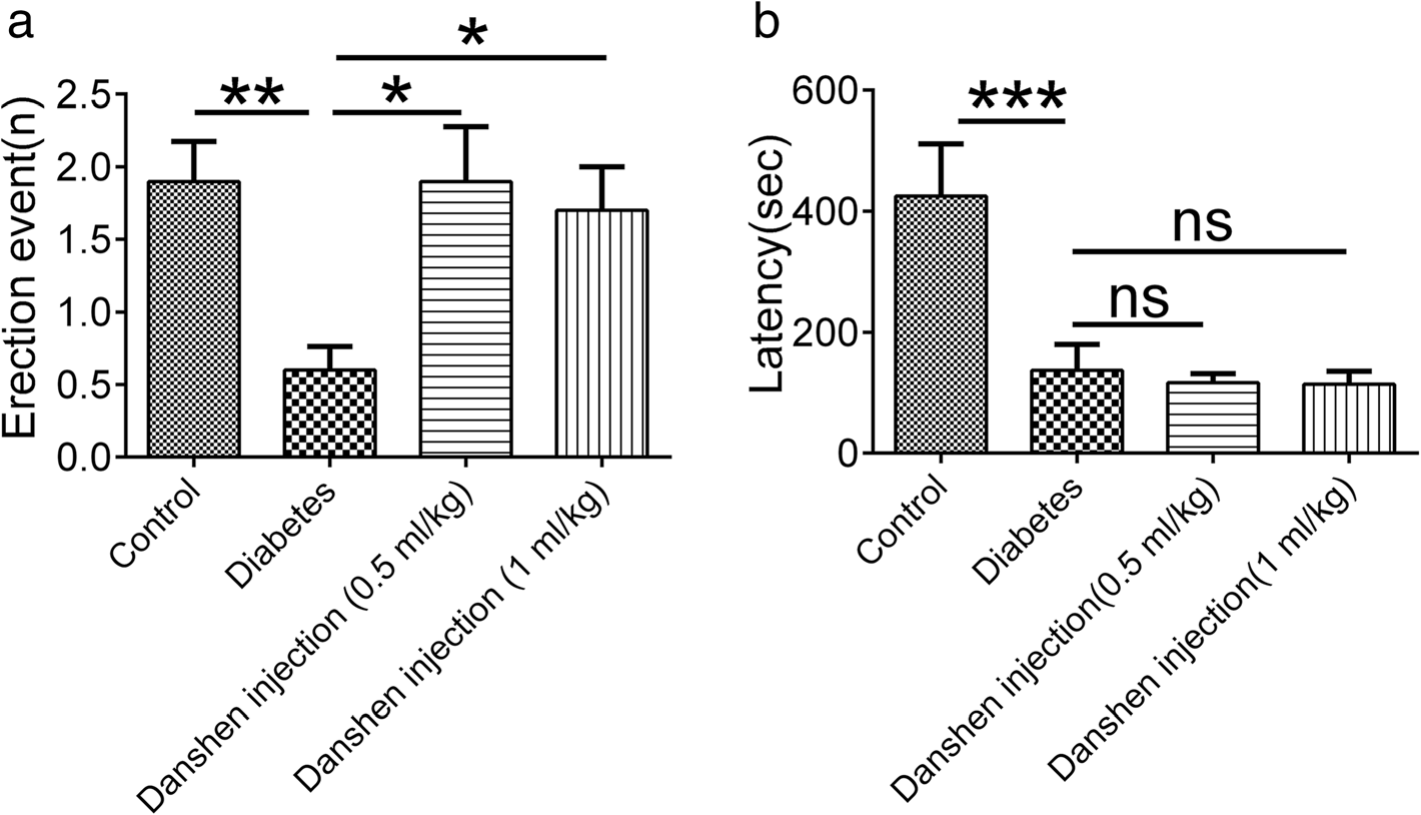
Danshen injection alleviated erectile dysfunction in diabetic rats. Treatment with Danshen injection for 6 weeks could improve erectile function via increasing erection times (**a**) but could not prolong the erection duration (**b**). Data are presented as the mean ± standard error of the mean (S.E.M.) for ten rats per group. *P < 0.05; ns, not significant

The mean times of erection episodes within 30 min were determined for evaluation of erectile function. The mean erection time was 1.70 ± 0.34 in the control group, and it was significantly reduced in the diabetes group (0.60 ± 0.16, *P* < 0.05). Meanwhile, Danshen injection (0.5 or 1 mL/kg) could improve erectile function by increasing the erection time (1.90 ± 0.38; 1.79 ± 0.30, Fig. 2a, *P* < 0.05), compared with the saline-treated diabetic rats. On the other hand, the erection duration was 425.00 ± 86.90 s in the control group and 137.14 ± 42.86 s in the diabetes group. However, Danshen injection (0.5 or 1 mL/kg) did not prolong the erection duration (116.50 ± 15.05 s; 114.07 ± 21.93 s, Fig. 2b, *P* > 0.05). These data suggested that Danshen injection could partially improve erectile function.

### Danshen injection ameliorated morphological abnormalities of penile tissue induced by hyperglycemia

**Fig. 3:**
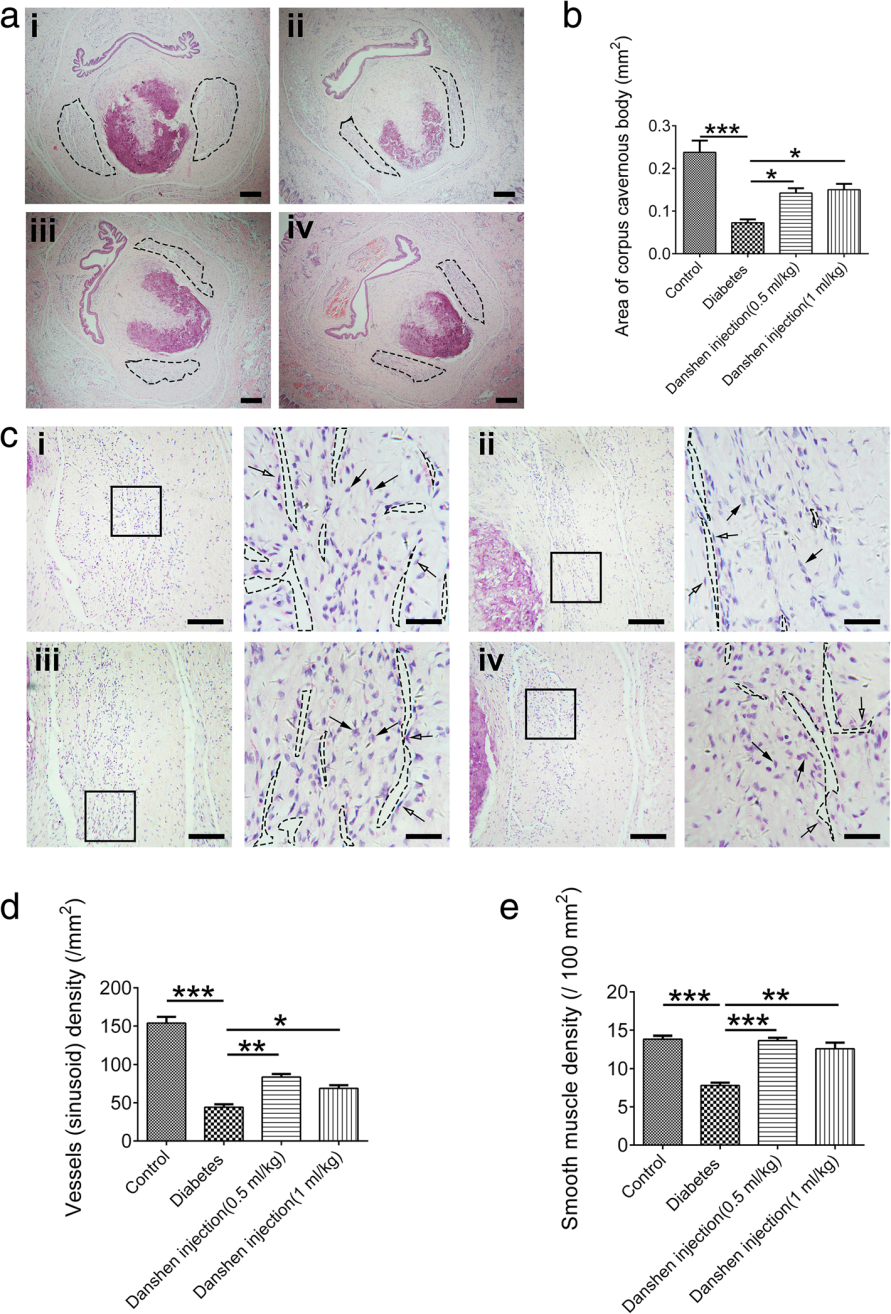
Danshen injection ameliorated morphological abnormalities of penile tissue in diabetic rats. **a** The morphology of penile tissue in normal rats exhibited a large, intact architecture of the corpus cavernous tissue, which is indicated by the dotted lines (i). There was significant shrinkage of the corpus cavernous body in the diabetes group (ii); however, the area of the corpus cavernous body was increased by Danshen injection (0.5 or 1 mL/kg) treatment (iii and iv). Scale bar: 200 μm. **b** Data represent the means ± S.E.M. for 10 sections of five rats per group. **c** Significant alterations of vessels (sinusoid) and smooth muscle cells in the corpus cavernous. The density of vessels, which are highlighted by the dotted lines, was surrounded by endothelial cells (indicated by the white arrow); and the number of smooth muscle cells (indicated by the black arrow) in the control group (i) was higher than that in the diabetes group (ii). Danshen injection (0.5 or 1 mL/kg) could rescue the reduction of vessel and smooth muscle cell density (iii, iv). The *inset* in each picture (Scale bar: 200 μm) is enlarged and displayed on the *right* (Scale bar: 50 μm). **d**, **e** Bar graphs represent the quantitative difference in the density of vessels (sinusoid) and smooth muscle cells. Data represent the means ± S.E.M. for 10 sections of five rats per group. *P < 0.05; **P < 0.01; ***P < 0.001

Morphological assessment of penile tissue was performed by hematoxylin-eosin staining. First, the corpus cavernous body was observed under low magnification. The rats in the control group exhibited a higher corpus cavernous area, which is indicated by the dotted line in Fig. 3ai; however, significant shrinkage of the corpus cavernosum body was observed in rats of the diabetes group (Fig 3aii). Meanwhile, administration of Danshen injection could rescue the reduction of the corpus cavernous (Fig. 3biii–iv and Fig. 3b). Second, vessels (sinusoid) in the corpus cavernous tissue, which are highlighted by the dotted lines, were surrounded by endothelial cells (indicated by the white arrow) (Fig. 3c). The vessel density (sinusoid) was 153.85 ± 8.26/mm^2^ in the control group (Fig. 3ci) and was significantly reduced in the diabetes group (44. 31 ± 3.76/mm^2^, Fig 3cii); however, treatment with Danshen injection (0.5 or 1 mL/kg) for 6 weeks could increase the vessel density (83.69 ± 4.02, 68.92 ± 4.18/mm^2^, respectively; Fig. 3ciii, iv and d). Third, the density of the smooth muscle cells, indicated by the black arrows, was also quantified. The results showed that a reduction of smooth muscle cells in diabetic rats could be rescued by application of Danshen injection (0.5 or 1 mL/kg) (Fig. 3e).

### Danshen injection downregulated Grp78 and CHOP expression as well as alleviated ER stress-induced apoptosis

**Fig. 4:**
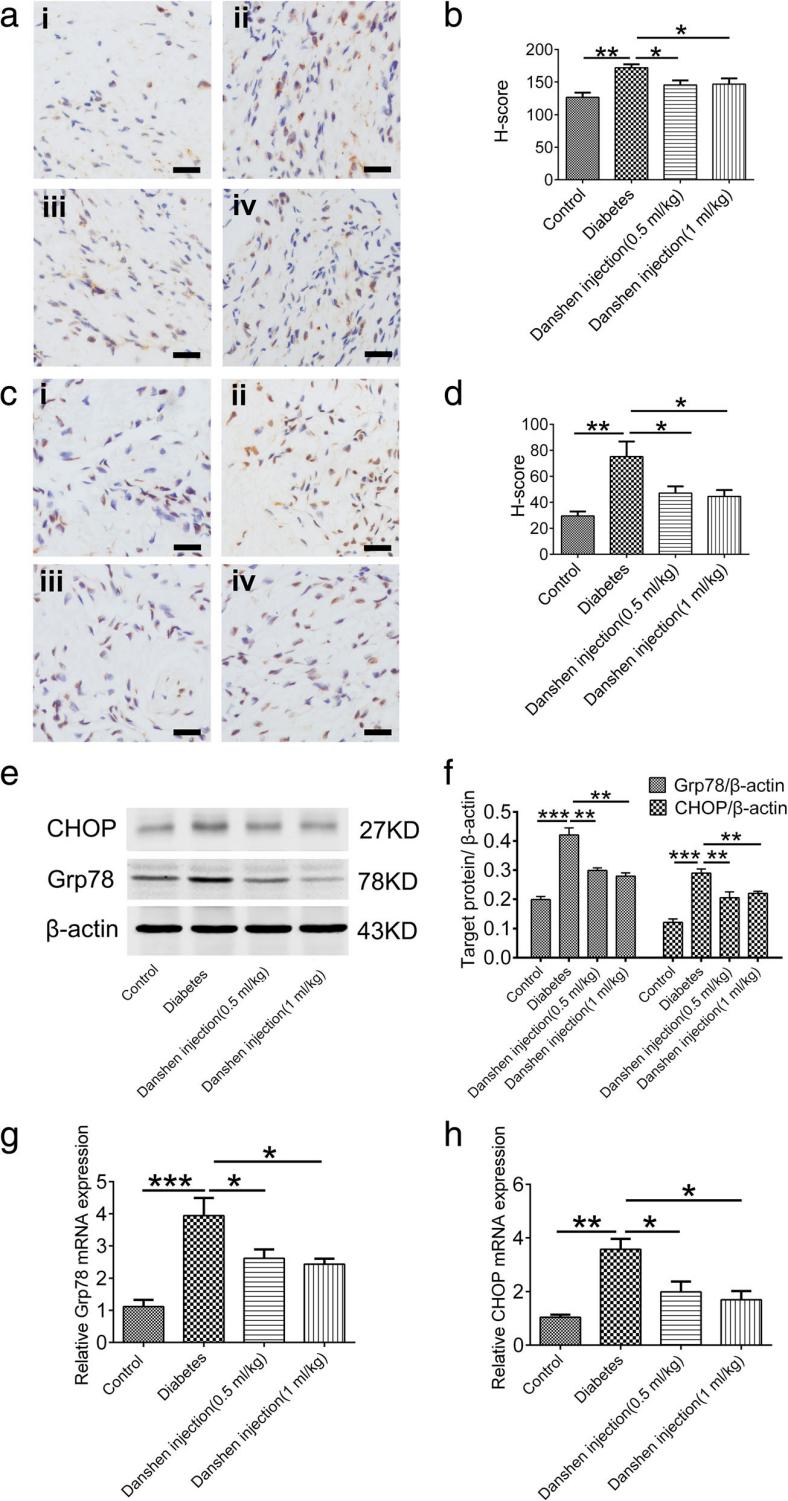
Danshen injection suppressed ER stress via downregulation of Grp78 and CHOP in corpus cavernous tissue. **a** Immunohistochemical analysis of Grp78 expression in penile tissue sections: (i) Control group; (ii) Diabetes group; (iii) Danshen injection (0.5 mL/kg) group; (iv) Danshen injection (1 mL/kg) group. **b** Data represent the means ± S.E.M. for five rats per group. Scale bar: 20 μm. **c** Immunohistochemical analysis of CHOP expression: (i) Control group; (ii) Diabetes group. (iii) Danshen treatment (0.5 mL/kg) group; (iv) Danshen treatment (1 mL/kg) group. **d** Data represent the means ± S.E.M. for five rats per group. Scale bar: 50 μm. **e** Western blot analysis of Grp78 and CHOP expression in penile tissue and (**f**) densitometry of the respective blots. Data are represented as the means ± S.E.M. for five rats per group, normalized to β-actin and expressed as a percentage of the control. **g**, **h** The mRNA levels of Grp78 and CHOP among the four groups (*n* = 5)

Grp78 is an ER chaperone that is expressed in response to an ER stress event, and its expression leads to ER stress-induced apoptosis via multiple pathways, accompanied by upregulation of CHOP, a proapoptic protein [20]. By immunohistochemical analysis, Grp78 was mainly expressed in the cytoplasm, while CHOP was predominately located in the nucleus (Fig. 4a and c). The H-scores of Grp78- and CHOP-positive areas were obviously increased in the cavernous tissue of saline-treated diabetic rats (171.98 ± 5.21 and 75.21 ± 11.62, respectively, Fig. 4aii and cii) in comparison with those in the control group (126.92 ± 7.09 and 29.55 ± 3.44, respectively, Fig. 4ai and ci). The increased H-score was reduced after Danshen injection at 0.5 mL/kg (Grp78: 145.40 ± 7.08; CHOP: 47.10 ± 5.25, *P* < 0.05, Fig. 4aiii and ciii) and at 1 mL/kg (Grp78: 146.82 ± 8.73; CHOP: 44.55 ± 4.90, *P* < 0.05, Fig. 4aiv and civ). Similar results were observed by immunoblotting analysis (Fig. 4e). In addition, the mRNA levels of Grp78 and CHOP were significantly increased in the diabetes group, compared with the controls (*P* < 0.05, Fig. 4f); whereas treatment with Danshen injection for 6 weeks could effectively decrease the expression levels of Grp78 and CHOP (*P* < 0.05 vs. the diabetes group, Fig. 4f).

**Fig. 5:**
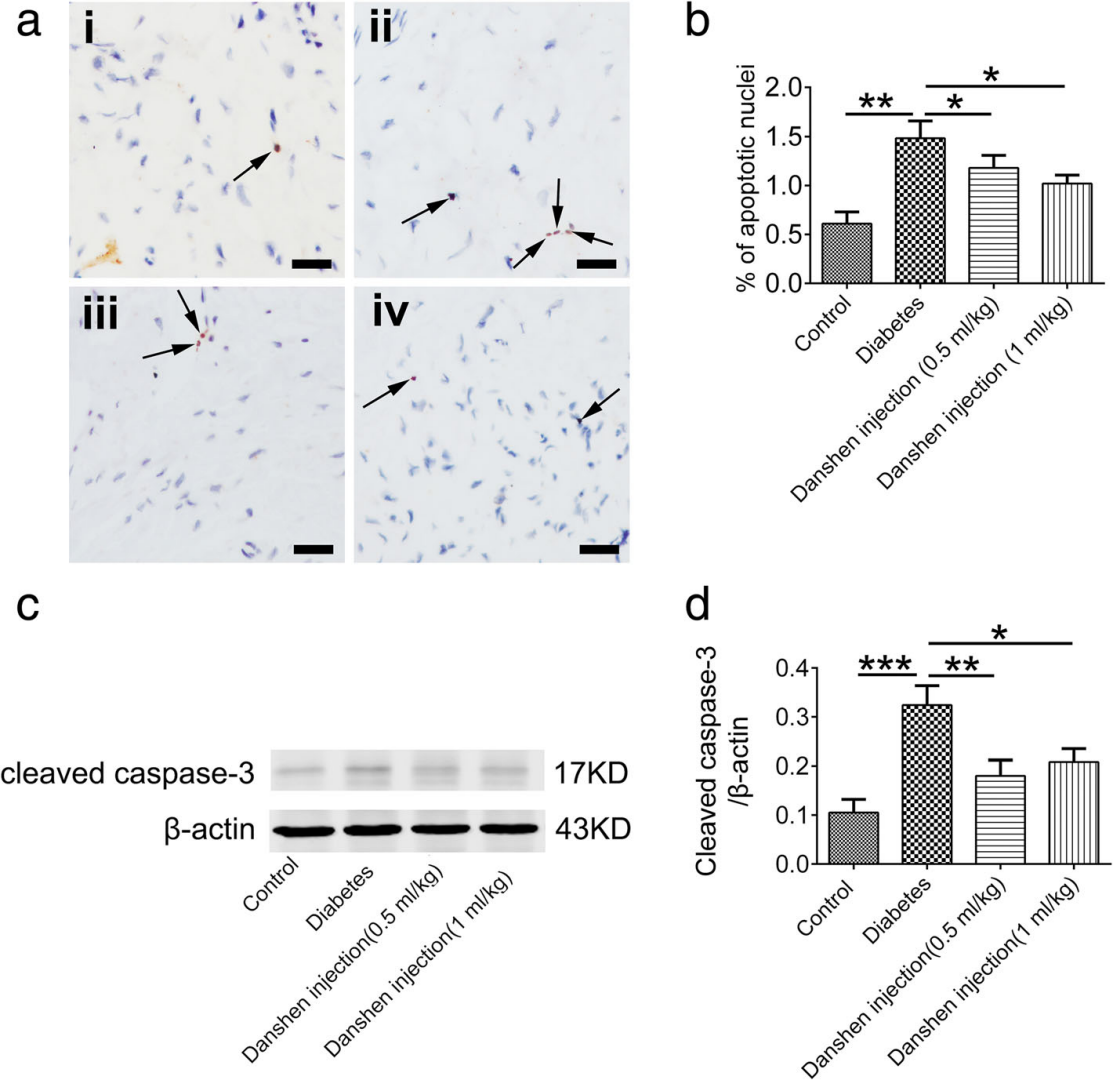
Danshen injection alleviated apoptosis. **a** Representative images of apoptotic cells in the corpus cavernous tissue by a TUNEL assay are shown. (i) Control group; (ii) Diabetes group; (iii) Danshen injection group (0.5 mL/kg); (iv) Danshen injection group (1 mL/kg). Hyperglycemia triggered an increase in the number of apoptotic cells (arrow), whereas Danshen injection potentially reduced the number of apoptotic cells. **b** Apoptotic nuclei were quantified. Data are presented as the mean ± standard error of the mean (S.E.M.). *P < 0.05; **P < 0.01. Scale bar: 20 μm. **c** The expression of cleaved caspase-3 was detected by western blot analysis. Cleaved caspase-3 was upregulated in the diabetes group, which was prevented by the Danshen injection. **d** Data are presented as the mean ± standard error of the mean (S.E.M.) for five rats per group. *P < 0.05; **P < 0.01

As mentioned above, CHOP plays an important role in cellular apoptosis. In order to determine whether Danshen injection could reduce apoptosis, a TUNEL assay was carried out to detect apoptotic cells. The percentages of apoptotic cells were 0.61 ± 0.12%, 1.48 ± 0.18%, 1.18 ± 0.13%, and 1.02 ± 0.09% (Fig. 5ai–iv, *P* < 0.05) in the cavernous tissues of control rats, saline-treated diabetic rats, and Danshen injection-treated diabetic rats (0.5 or 1 mL/kg), respectively, suggesting that hyperglycemia triggers an increase in apoptotic cells, whereas Danshen injection had a potentially beneficial effect on suppressing ER stress-induced apoptosis (Fig. 5b, *P* < 0.05). Furthermore, the expression of cleaved caspase-3 was reduced by treatment with Danshen injection (Fig. 5b).

### Danshen injection reduced ROS levels via increased SOD activity

Oxidative stress is considered as a major factor triggering ER stress. In this study, the effect of Danshen injection on oxidative stress was examined. The diabetic rats exhibited increased ROS activity in cavernous tissue homogenates as compared with the normal rats (presented as 2′,7′-dichlorofluorescein fluorescence intensity, 164.53 ± 8.71% of the control, *P* < 0.01, Table 1). The increase in ROS activity was partially reversed by Danshen injection at 0.5 mL/kg and at 1 mL/kg (130.62 ± 7.88%, 122.17 ± 9.26% of the control, respectively; *P* < 0.05 vs. the diabetes group), although it was still higher than that in the control group (*P* < 0.01 vs. the control group). Similar trends were found for another oxidative stress marker, MDA (control group, 1.41 ± 0.09 nmol/mg of protein; diabetes group, 2.22 ± 0.09 nmol/mg of protein; Danshen injection group with 0.5 mL/kg, 1.80 ± 0.10 nmol/mg of protein; Danshen injection group with 1 mL/kg, 1.79 ± 0.08 nmol/mg of protein; Table 1). On the contrary, Danshen treatment at both 0.5 mL/kg and 1 mL/kg increased the activity of SOD, an antioxidant enzyme, in cavernous tissue homogenates (222.98 ± 6.71 U/mg of protein and 228.77 ± 7.11 U/mg of protein, respectively), which was decreased in the diabetic rats (191.24 ± 4.87 U/mg of protein). These data suggested that Danshen treatment as an antioxidant agent could suppress the level of hyperglycemia-induced oxidative stress.

**Table 1:** Oxidative stress indicators in rat penile tissue

Group	ROS	MDA content (nmol/mg of protein)	SOD activity (U/mg of protein)
Control	99.26 ± 0.89	1.41 ± 0.09	267.14 ± 15.98
Diabetes	164.53 ± 8.71^**^	2.22 ± 0.09^**^	191.24 ± 4.87^***^
Danshen injection (0.5 mL/kg)	130.62 ± 7.88^*#^	1.80 ± 0.10^**#^	222.98 ± 6.71^**#^
Danshen injection (1 mL/kg)	122.17 ± 9.26^*#^	1.79 ± 0.08^*#^	228.77 ± 7.11^**#^

Data are presented as the mean ± S.E.M. (n = 5). **P < 0.01, ***P < 0.001 vs. the control group; #*P* < 0.05, vs. the diabetes group; ROS data are presented as 2′,7′-dichlorofluorescein fluorescence intensity (% of control)

*ROS* Reactive oxygen species, *MDA* Malondialdehyde, *SOD* Superoxide dismutase

## Discussion

Traditional Chinese herbal decoctions have been used as a unique curative method for the treatment of diabetic ED [21]. According to our previous study and other reports, Danshen injection can attenuate renal injury in animal models of diabetes [17, 22]. It is known that renal dysfunction is indeed a risk factor for the induction of ED, though the underlying mechanism is not fully elucidated [23, 24]. In fact, a large number of factors have been proposed to be involved, such as hormonal abnormalities, endothelial dysfunction, and disturbance of the autonomic nervous system in renal tissue [25]. Therefore, we believe that renal injury is definitely related to ED. However, no studies have investigated the beneficial effect of Danshen injection on hyperglycemia-induced ED. In the present study, we found that Danshen injection could alleviate ultrastructural changes of cavernous tissue and rescue erectile function in a STZ-induced diabetic rat model via inhibition of ER stress-induced apoptosis.

In fact, ER stress-induced apoptosis has been found in various organs of diabetes models, including the brain, renal tissue, and cardiomyocytes [14, 26, 27]. Accumulating evidence has demonstrated the involvement of multiple factors to trigger the activation of ER stress under hyperglycemia, among which oxidative stress is considered as the most prominent factor [28]. It is well known that disulfide bond formation and proper protein folding can be disrupted by oxidative stress via disturbance of the ER redox state, accompanied by an elevated production of ROS. ROS refer to chemical species that are generated upon incomplete reduction of oxygen. In this study, ROS production was significantly increased in the cavernous tissue of diabetic rats, and these findings were consistent with those of a previous study [29]. Interestingly, Danshen injection could reduce ROS production in a setting of diabetes. Our team and other investigators have found that Danshen injection, as a potent scavenger of superoxide radicals, can alleviate oxidative stress and reduce the production of ROS via increasing SOD activity in renal tissue of STZ-induced diabetic rats [17, 22]. Therefore, we speculated that the mechanism by which Danshen injection exerts its protective effect on cavernous tissue against oxidative stress is probably through the increase of SOD activity. Consistent with this hypothesis, our results showed that the SOD activity was elevated in the Danshen injection-treated diabetic rats, accompanied by a decreased content of MDA, a terminal product of lipid peroxidation, revealing an alleviated degree of oxidative stress in the Danshen injection-treated diabetic rats [30]. Taken together, Danshen injection could attenuate oxidative stress through increasing SOD activity.

As mentioned previously, prolonged oxidative stress leads to ER dysfunction and activation of ER stress. With the accumulation of unfolded or misfolded proteins in the ER, Grp78 (also known as 78-kDa glucose-regulated protein), which exists in the ER membrane, is attracted to bind to unfolded proteins and aid correct protein folding. Subsequently, Grp78 is released from three ER stress sensors, namely pancreatic ER kinase-like ER kinase (PERK), activating transcription factor 6 (ATF6), and inositol-requiring enzyme 1 (IRE1), thereby activating ER stress [31]. If this activation is sustained, all three sensors can induce the expression of growth arrest and DNA damage-inducible gene 153 (GADD153/CHOP), which is regarded as a characteristic biomarker of ER stress-induced apoptosis. In this study, both Grp78 and CHOP were upregulated in the cavernous tissue of diabetic rats. However, Danshen injection could reverse the increased expression of these two proteins. This process was accompanied by an increase of SOD activity and a reduction of MDA content. These data suggested that Danshen injection may suppress oxidative stress-induced ER stress in the cavernous tissue of a STZ-induced diabetic rat model. Furthermore, apoptosis is a critical factor involved in the pathophysiology of ED [32]. In this study, the percentage of apoptotic cells detected by a TUNEL assay and the expression of activated caspase-3 were increased in the diabetic rats, and Danshen injection could significantly reduce the number of apoptotic cells in the cavernous tissues of the diabetic rats. We also observed reduced erection times in the diabetic rats, which were improved by treatment with Danshen injection. We speculated that this beneficial effect of Danshen injection on rescuing erectile function might be associated with inhibition of ER stress-induced apoptosis. However, the determination of which ER stress sensor or pathway that is responsible for the upregulation of CHOP was not explored in this study. Based on previous findings, PERK signaling is sustained in the proapoptotic phase, whereas the IRE1α–XBP1 and ATF6 pathways are turned off in cells undergoing prolonged ER stress [33]. Therefore, we hypothesized that the induction of CHOP expression in diabetic ED rats was through the PERK pathway. But confirmation of this hypothesis requires further work in the future.

On the other hand, the morphological change of cavernous tissue was another consequence of ED. It has been demonstrated that the loss of corporal smooth muscle cells in the corpus cavernous is one of most important characteristics of ED in diabetes patients [34, 35]. In this study, the shrinkage of the corpus cavernous tissue with a reduced smooth muscle density was observed in the diabetic rats, although the mechanism was not fully elucidated. Accumulating evidence has shown that oxidative stress may play an important role in the pathological mechanism of ED in diabetes patients [36]. In addition, the generation of ROS under conditions of chronic hyperglycemia may impair the synthesis of endothelial nitric oxide synthase and promote protein oxidation and endothelial apoptosis, resulting in inhibition of cavernous vascularization or relaxation of smooth muscle [1]. Consistently, our results showed apoptotic cells and reduced vessel density in the diabetic rats. Danshen injection could attenuate the morphological abnormalities via inhibiting ROS production and decreasing the oxidative stress level. The increased levels of antioxidants, such as SOD, can lead to reduced ROS levels. Moreover, accumulation of unfolded/misfolded proteins can also increase ROS production [37]. Therefore, ER stress and oxidative stress accentuate each other in a positive feedback manner [38]. Thus, Danshen injection could inhibit the activation of ER stress by reducing the numbers of apoptotic cells as well as alleviating oxidative stress.

## Conclusion

The present study reveals that ER stress-induced apoptosis accompanied by increased ROS production play an important role in the pathogenesis of ED in a STZ-induced diabetic rat model. Danshen injection could protect erectile function against oxidative stress and ER stress by increasing SOD activity.

## Abbreviations

DM: Diabetes mellitus
ED: Erectile dysfunction
ER: Endoplasmic reticulum
GADD153/CHOP: Growth arrest and DNA damage-inducible gene 153
Grp78: Glucose-regulated protein 78
MDA: Malondialdehyde
ROS: Reactive oxygen species
SOD: superoxide dismutase
